# Psychological well-being over time among adults with diabetes: a cross-sectional study

**DOI:** 10.1007/s00592-025-02461-y

**Published:** 2025-04-22

**Authors:** Dennis Wesselbaum

**Affiliations:** Department of Economics, University of Otago, Dunedin, New Zealand

**Keywords:** BRFSS, Diabetes mellitus, Well-being

## Abstract

This study examines the relationship between diabetes duration and well-being using data from 115,039 U.S. adults (2005–2017). Logistic regression shows a significant negative correlation, with each year of diabetes reducing well-being by 0.05%. This effect is stronger in men (3.7%) than women (1.9%), highlighting the need for psychological management in diabetes care.

**Supplementary Information:**

The online version contains supplementary material available at 10.1007/s00592-025-02461-y.

## Introduction

In 2019, diabetes mellitus ranked as the 8th highest contributor to global mortality and disability-adjusted life years (DALYs). Given its widespread prevalence and associated complications like cardiovascular and kidney diseases, diabetes poses significant burdens of both mortality and morbidity. It is projected to incur costs amounting to nearly 2% of the global gross domestic product (GDP). Research has shown that individuals living with diabetes experience lower psychological well-being compared to the general population.

While the impact of diabetes on psychological well-being is well-documented, the specific role of diabetes duration in shaping well-being over time remains underexplored. Existing studies have primarily focused on the general association between diabetes and mental health, without addressing how the length of time living with the disease influences this relationship. It is plausible that well-being fluctuates over time due to various factors, such as changes in physical health, emotional stress related to managing the disease and its uncertainties (e.g., risk of complications), financial strain, and lifestyle adjustments required for disease management (e.g., dietary restrictions and blood sugar monitoring). Furthermore, gender differences in this association have not been adequately investigated. This study fills this gap by examining how the duration of living with diabetes affects psychological well-being, with a particular focus on gender variations. The aim of the study is to provide a more nuanced understanding of how well-being is impacted by the duration of diabetes, exploring whether this effect differs between men and women. By addressing these gaps, the findings offer valuable insights for diabetes management, emphasizing the need for psychological support alongside medical treatment to improve well-being and treatment adherence [1, 2].

## Research design and methods

### Study population

I examine data from the *Behavioral Risk Factor Surveillance System* (BRFSS), a nationally representative telephone survey conducted by the *Centers for Disease Control and Prevention* (CDC) across all U.S. States, the District of Columbia, and three territories from 2005 to 2017. The analysis in this paper utilizes information from the “Diabetes” and “Emotional Support and Life Satisfaction” modules, supplemented with well-being covariates from other survey modules. Previous studies have confirmed the BRFSS’s reliability and validity and used it for analysing the relation between diabetes and psychological well-being.

### Key variables

The key outcome is well-being and is measured using the question “*In general*,* how satisfied are you with your life?*”. Life satisfaction is a crucial component of subjective well-being, as it encompasses assessments of various life aspects as well as life overall. A limitation of using ordinal data is that it does not provide information about the distance between response categories [3]. Since ordered categories only indicate rank rather than the actual interval between them, traditional methods may yield inaccurate estimates. To address this, I follow [3] and create a dummy variable for high well-being which is one if the respondent answers “very satisfied” or “satisfied” and zero if the response is “dissatisfied” or “very dissatisfied”. The main variable of interest is the duration a person lived with diabetes. I use the response to the question “*How old were you when you were told you have diabetes?*” and use the age variable to construct the duration. Finally, I include the standard confounding factors used in well-being analyses based on the related literature [4, 5] and established well-being frameworks (see Supplementary Material for a full description).

### Statistical analysis

I rely on descriptive statistics and logistic regressions to study the association between duration of diabetes and well-being. Formally, I estimate the following model

1 $$\:{WB}_{i,t}=\:\alpha\:+{\beta\:Duration}_{i,t}+\:\gamma\:{X}_{i,t}+{\delta\:}_{j}+{\delta\:}_{t}+\:{\epsilon\:}_{i,t}$$

where $\:{WB}_{i,t}$ is a binary variable indicating whether individual *i* in survey year *t* has high well-being or not. Control variables are included, $\:{X}_{i,t}$, and so are state, $\:{\phi\:}_{j}$, and year, $\:{\mu\:}_{t}$, fixed effects to account for time-invariant differences across states (e.g., policies) and time-varying confounders (e.g., business cycles and survey context effects). I use cluster-robust standard errors at the state-level and all *p*-values are two-sided (I use Stata SE18.0).

## Results

### Sample characteristics

In the BRFSS sample (*N* = 115,039), the prevalence of diabetes (types 1 and 2) increases from 10% (Men: 9.8%, women: 10.2%) in 2005 to 14% (Men: 13.6%, women: 14.4%) in 2017. Living with diabetes reduces well-being (*p* <.01) and this association is stronger for men compared to women (*p* <.01). The characteristics of my sample are presented in Table 1. On average, people lived for 10.05 ± 9.86 years with diabetes (Figure S2 in the Supplementary Material shows the distribution) and 91.1% ± 28.5% have high well-being.

**Table 1:** Descriptive statistics (*N* = 115,039)

	Mean	Std. Dev.	Min	Max
High well-being	0.911	0.285	0	1
Diabetes: duration	10.048	9.859	0	78
Diabetes: education	0.595	0.491	0	1
Diabetes: doctor visits	4.408	5.471	0	76
Male	0.529	0.499	0	1
Age	57.152	12.751	18	79
Ethnicity				
White	0.676		0	1
Black	0.153		0	1
Asian	0.018		0	1
Hispanic	0.111		0	1
Other	0.042		0	1
Educational status				
Some High School	0.144		0	1
High school and some college	0.604		0	1
College	0.252		0	1
Marital status				
Married or Partner	0.656		0	1
Divorced or Separated	0.156		0	1
Widowed	0.100		0	1
Single	0.089		0	1
Child	0.242	0.428	0	1
General health	0.547	0.498	0	1
Mental health	0.160	0.307	0	1
Chronic diseases	0.370	0.483	0	1
Smoker	0.169	0.374	0	1
Exercise	0.638	0.481	0	1
Social capital	0.741	0.438	0	1
Employment	0.420	0.494	0	1
Income group				
< $20,000	0.259		0	1
≥ $20,000 and <$50,000	0.402		0	1
≥ $50,000	0.339		0	1

BRFSS sample weights applied. Observations are 115,039 for all variables.

### Regression results

The association between the duration of diabetes and high well-being are presented in Table 2. I find that with every year lived with diabetes, the likelihood of high well-being is significantly lower (*p* <.01). Living 80 years with diabetes compared to just being diagnosed with diabetes results in the likelihood of high well-being being between about 3 and 5% points lower depending on whether health controls are included or not. These results vary across sex at birth: I find a significantly stronger association for men compared to women (3.7% vs. 1.9% for the same comparison, *p* <.01).

The findings concerning the control variables (refer to Supplementary Material Table S.1) align with previous research [4, 5]. In summary, high income, good physical and mental health, social support, and being married or having a partner are all positively associated with high well-being.

**Table 2:** Regression results

	High well-being			Male
	Full Sample	Full Sample	Female	
Duration of diabetes	-0.0004***	-0.0002***	-0.0001*	-0.0002**
	(0.0001)	(0.0001)	(0.0001)	(0.0001)
Controls	Yes	Yes	Yes	Yes
Health controls	No	Yes	Yes	Yes
Obs.	115,039	114,550	65,606	48,944
Pseudo R^2^	0.293	0.313	0.320	0.315

The table shows average marginal effects from a logit regression with well-being as the dependent variable. Control estimates are in Table S.1 of the supplementary material. All regressions include fixed effects for year and state, with standard errors clustered at the state-level. Significance levels: ***p <.01, **:*p* <.05, *:*p* <.10.

## Discussion

In my cohort of adults diagnosed with both type 1 and type 2 diabetes, I observe a negative correlation between well-being and the duration of living with the condition, even after accounting for various confounding factors. This association is stronger for men compared to women.

The gender differences in the well-being-duration relationship warrant further attention. While both men and women experience a decline in well-being as the duration of their diabetes increases, the impact appears more pronounced for men. This may be related to different social and psychological coping mechanisms between the sexes, as well as potential gender-specific stressors. For instance, research suggests that men may be less likely to seek emotional support or utilize mental health resources compared to women, which could contribute to a more significant decline in their well-being over time. Additionally, the societal expectations placed on men to maintain independence and manage challenges without external help may exacerbate the psychological burden of living with a chronic condition like diabetes.

I explored additional implications of my results, discussing them as exploratory findings. My analysis reveals that neither attending diabetes management classes nor frequent doctor visits (more than once per month on average) impact the association between well-being and the duration of diabetes. This observation applies to both men and women. Furthermore, I do not find evidence that age at the diagnosis interacts with the well-being-duration relationship.

My novel findings carry significant practical implications. While a diabetes diagnosis necessitates a medical treatment plan and education on self-management, it also warrants a psychological management strategy to mitigate adverse impacts on well-being. Furthermore, my findings suggest that as the disease progresses, the importance of psychological management intensifies, as potential negative interferences with treatment adherence may escalate over time [1, 2]. Examining the impact of diabetes on well-being over time offers a comprehensive view of its influence on quality of life. My research offers fresh perspectives valuable for advancing well-being, aligning with the objectives outlined in United Nations Sustainable Development Goal 3.4.

I note several limitations. Due to the cross-sectional design, causation cannot be inferred as I lack an external instrument for directionality. Additional factors like treatment details, associated with both well-being and diabetes duration, are not included in my study. Similarly, while I control for various health outcomes, complications of diabetes could affect well-being for which I again lack data. Finally, the relationship may vary by type of diabetes, which is not available in the data.

## Electronic supplementary material

Below is the link to the electronic supplementary material.

Supplementary Material 1.
