# Supplementary material for: Psychological well-being over time among adults with diabetes: a cross-sectional study
Source: Acta Diabetol. 2025 Apr 22;62(5):773–6. doi: 10.1007/s00592-025-02461-y (PMC12116984; doi:10.1007/s00592-025-02461-y)
Supplement: Supplementary file 1 — Supplementary Material 1. [file 592_2025_2461_MOESM1_ESM.docx]

**Supplementary Material**

S.1 Conceptual Framework

The statistical model we chose and the covariates we incorporated in our research are guided by the theoretical frameworks established by [18,19]. An outline of this framework is provided in Figure S.1. The framework suggests that subjective well-being is influenced by factors such as demographics, living conditions, physical and mental health, and social networks. Each of these broad categories encompasses numerous elements that could potentially impact well-being. For instance, aspects like income and job status can shape an individual’s living conditions. We recognize that these factors will interact with each other, resulting in mediating and moderating effects that are not depicted in the figure.

Figure S.1: Conceptual Framework (adapted from [18,19])

S.2 Control Variables

We begin with standard demographic factors. We use the imputed age variable provided by the BRFSS. This variable is collapsed above 80 and we therefore restrict our sample to those below 80 years of age to avoid measurement error. A binary variable is created to represent male respondents. Ethnicity is captured by a five-level categorical variable (White, Black, Asian, Hispanic, Other). Education is classified into three tiers: some high school (including those who never attended, elementary, or completed some high school), high school and some college, and college. Marital status encompasses four categories: married/partnered, divorced/separated, widowed, and single (never married). Additionally, a dummy variable is generated to indicate whether respondents have children. General health is captured through respondents' self-assessment, categorized as excellent, very good, good, fair, or poor. The dummy variable is set to one for responses indicating good health or better, and zero otherwise. Another dummy variable is created to identify respondents who self-report having one of the following chronical diseases: hear attack, angina, stroke, or asthma. Exercise participation is represented by a binary variable, set to one for those engaging in physical activities outside of their regular job in the past month, and zero otherwise. Mental health is measured by using the response to the following question: “*Now thinking about your mental health, which includes stress, depression, and problems with emotions, for how many days during the past 30 days was your mental health not good?*”. We furthermore include a control for smoking behavior. Smoking is represented by a dummy variable, with a value of one if the respondent reports being a current smoker (either daily or occasionally) and zero if they indicate never having smoked or being a former smoker. Social capital is gauged by respondents' access to emotional support, with a dummy variable set to one for those reporting always or usually receiving support, and zero otherwise. Employment status is captured through a dummy variable, set to one for respondents who are employed or self-employed, and zero for those unemployed, homemakers, students, or retired. Household income is categorized into three levels: less than $20,000, $20,000 to less than $50,000, and $50,000 or more. The Metropolitan Statistical Area (MSA) variable categorizes areas based on their geographical location and urbanization level within a broader metropolitan region. The variable has four levels: center city of an MSA, Outside the center city of an MSA but inside the county containing the center city, Inside a suburban county of the MSA, MSA without center city, and and not in an MSA. We incorporate two variables pertaining to diabetes management. Firstly, we include a variable indicating whether the respondent has received education on managing diabetes. Utilizing the query "*Have you ever taken a course or class on how to manage your diabetes yourself?*", we generate a dummy variable assigned a value of one for a "yes" response and zero for a "no" response. Secondly, we introduce a variable capturing the frequency of the respondent's doctor visits. Using the question "*About how many times in the past 12 months have you seen a doctor, nurse, or other health professional for your diabetes?*", we create a dummy variable assigned a value of one for responses exceeding 12 visits (more than one visit per month, on average), and zero otherwise.

S.3 Additional Tables and Figures

Figure S.2: Histogram of Diabetes Duration (in years)


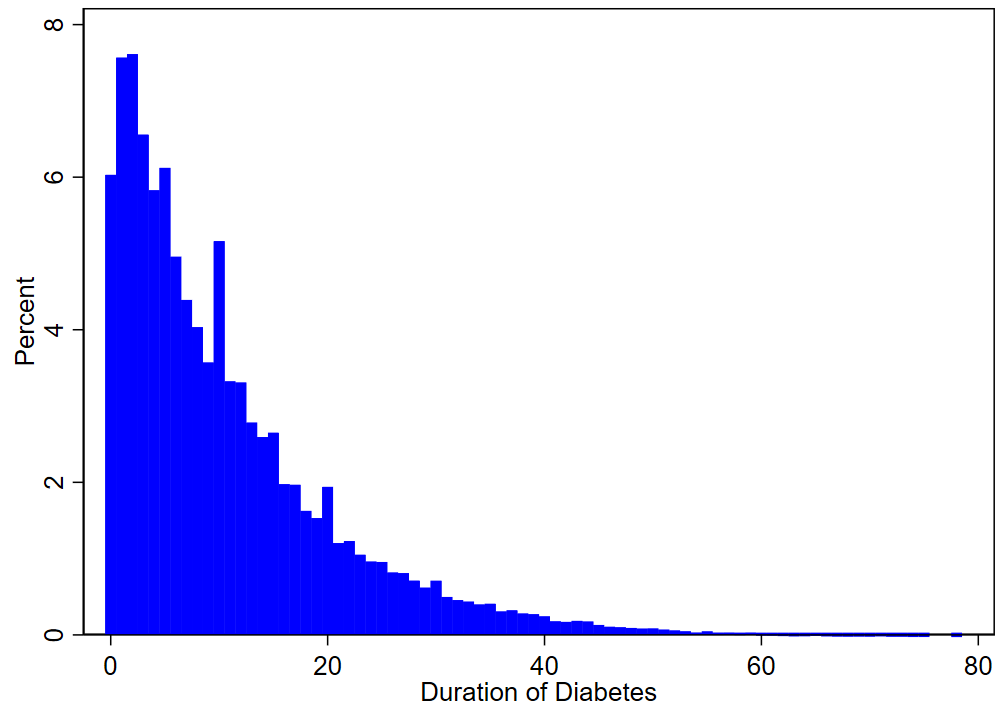


Table S.1: Results for Control Variables

|  | (1) | (2) | (3) | (4) |
| --- | --- | --- | --- | --- |
| Diabetes: Education | 0.085^**^ | 0.038 | -0.043 | 0.126^**^ |
|  | (0.041) | (0.041) | (0.089) | (0.054) |
| Diabetes: Doctor Visits | -0.253^**^ | -0.137 | -0.152 | -0.143 |
|  | (0.124) | (0.125) | (0.167) | (0.226) |
| Age | -0.079^***^ | -0.059^***^ | -0.040^*^ | -0.079^***^ |
|  | (0.015) | (0.015) | (0.024) | (0.016) |
| Age Squared | 0.001^***^ | 0.001^***^ | 0.001^**^ | 0.001^***^ |
|  | (0.000) | (0.000) | (0.000) | (0.000) |
| *Ethnicity* |  |  |  |  |
| Black | 0.412^***^ | 0.382^***^ | 0.347^***^ | 0.403^***^ |
|  | (0.102) | (0.098) | (0.130) | (0.121) |
| Asian | 0.996^***^ | 0.917^***^ | 0.940 | 1.076^***^ |
|  | (0.207) | (0.213) | (0.639) | (0.411) |
| Hispanic | 0.483^***^ | 0.519^***^ | 0.270^***^ | 0.774^***^ |
|  | (0.064) | (0.070) | (0.102) | (0.164) |
| Other | 0.022 | 0.067 | 0.028 | 0.112 |
|  | (0.123) | (0.126) | (0.133) | (0.207) |
| *Educational Status* |  |  |  |  |
| Some High School | 0.142 | 0.331^***^ | 0.495^***^ | 0.200 |
|  | (0.090) | (0.087) | (0.093) | (0.142) |
| High School and College | 0.129^**^ | 0.228^***^ | 0.346^***^ | 0.143^*^ |
|  | (0.051) | (0.050) | (0.072) | (0.077) |
| *Marital Status* |  |  |  |  |
| Married or Partner | 0.286^***^ | 0.315^***^ | 0.320^**^ | 0.254^**^ |
|  | (0.085) | (0.083) | (0.136) | (0.111) |
| Divorced or Separated | -0.120 | -0.069 | -0.093 | -0.081 |
|  | (0.082) | (0.082) | (0.110) | (0.139) |
| Widowed | -0.024 | -0.006 | 0.109 | -0.189 |
|  | (0.096) | (0.094) | (0.103) | (0.157) |
| Child | 0.091 | 0.109 | 0.094 | 0.147^*^ |
|  | (0.073) | (0.073) | (0.085) | (0.089) |
| Employment | 0.641^***^ | 0.447^***^ | 0.446^***^ | 0.441^***^ |
|  | (0.051) | (0.057) | (0.099) | (0.062) |
| *Income Group* |  |  |  |  |
| >$20,000 - <$50,000 | 0.341^***^ | 0.253^***^ | 0.324^***^ | 0.158^**^ |
|  | (0.070) | (0.066) | (0.107) | (0.070) |
| >$50,000 | 0.765^***^ | 0.577^***^ | 0.520^***^ | 0.609^***^ |
|  | (0.063) | (0.063) | (0.098) | (0.088) |
| Social Capital | 1.675^***^ | 1.609^***^ | 1.740^***^ | 1.504^***^ |
|  | (0.047) | (0.046) | (0.048) | (0.066) |
| Mental Health | -2.129^***^ | -1.881^***^ | -1.853^***^ | -1.924^***^ |
|  | (0.061) | (0.060) | (0.070) | (0.100) |
| General Health |  | 0.879^***^ | 0.752^***^ | 1.018^***^ |
|  |  | (0.050) | (0.079) | (0.090) |
| Chronic Diseases |  | -0.112^***^ | -0.190^***^ | -0.047 |
|  |  | (0.037) | (0.042) | (0.070) |
| Exercise |  | 0.358^***^ | 0.433^***^ | 0.295^***^ |
|  |  | (0.037) | (0.061) | (0.062) |
| Smoker |  | -0.203^***^ | -0.294^***^ | -0.139 |
|  |  | (0.057) | (0.064) | (0.088) |
| Obs. | 115,039 | 114,550 | 65,606 | 48,944 |

Notes: Dependent variable in all regression is high well-being. Results for MSAs not shown. All regressions include fixed effects for year and state, with standard errors clustered at the state-level. Significance levels: ***: *p* < .01, **: *p* < .05, *: *p* < .10.
